# Circulating Claudin-5 and Systemic Inflammatory Indices in Wet and Dry Age-Related Macular Degeneration

**DOI:** 10.3390/medicina62050823

**Published:** 2026-04-26

**Authors:** Onur Çatak, Jülide Kurt Keleş, Zekiye Çatak

**Affiliations:** 1 Department of Ophtalmology, Faculty of Medicine, Firat University, 23119 Elazig, Turkey; 2 Department of Ophtalmology, Fethi Sekin City Hospital, Health Sciences University, 23280 Elazig, Turkey; 3 Department of Medical Biochemistry, Fethi Sekin City Hospital, Health Sciences University, 23040 Elazig, Turkey

**Keywords:** tight junction protein, blood-retinal barrier dysfunction, hemogram-derived inflammatory indices, platelet-to-lymphocyte ratio, visual function, retina

## Abstract

*Background and Objectives*: Age-related macular degeneration (AMD) is a multifactorial retinal disease in which inflammation and blood-retinal barrier dysfunction may contribute to disease pathogenesis. Claudin-5 is a key tight-junction protein involved in endothelial barrier integrity. Hemogram-derived indices such as the neutrophil-to-lymphocyte ratio (NLR), platelet-to-lymphocyte ratio (PLR), monocyte-to-lymphocyte ratio (MLR), and pan-immune-inflammation value (PIV) reflect systemic inflammatory status. This study aimed to evaluate circulating claudin-5 levels and systemic inflammatory indices in patients with wet and dry AMD and to investigate their associations with visual function. *Materials and Methods*: This prospective case–control study included 90 participants: 30 patients with wet AMD, 30 patients with dry AMD, and 30 healthy controls. All participants underwent detailed ophthalmologic examination, including best-corrected visual acuity (BCVA) assessment and optical coherence tomography. Serum claudin-5 levels were analyzed by enzyme-linked immunosorbent assay, and NLR, PLR, MLR, and PIV were calculated from complete blood count parameters. Group comparisons, correlation analyses, and age-adjusted analyses were performed using appropriate statistical methods. *Results*: Age differed significantly among the groups (*p* = 0.032), with the highest median age in the dry AMD group. BCVA (logMAR) also differed significantly (*p* < 0.001), and both AMD groups had worse visual acuity than controls. Median serum claudin-5 levels were 2.42 in controls, 3.28 in the wet AMD group, and 3.10 in the dry AMD group, with no significant between-group difference (*p* = 0.280). NLR, MLR, and PIV were also comparable among the groups (*p* = 0.310, *p* = 0.410, and *p* = 0.752, respectively). PLR differed among the groups (*p* = 0.019), and post hoc analysis showed higher PLR values in the dry AMD group than in the wet AMD group (*p* = 0.013). However, this difference was no longer statistically significant after adjustment for age (adjusted *p* = 0.098). Serum claudin-5 was not significantly correlated with age, BCVA, NLR, PLR, MLR, or PIV. *Conclusions*: Circulating claudin-5 did not differ significantly across AMD phenotypes and was not associated with age, visual function, or systemic inflammatory indices. Although PLR differed between wet and dry AMD before adjustment for age, the overall findings suggest that single-point peripheral serum measurements of claudin-5 may have limited utility in reflecting local retinal barrier-related changes in AMD. Larger longitudinal studies are needed to clarify its potential biomarker role.

## 1. Introduction

Age-related macular degeneration (AMD) is a major cause of permanent central visual impairment in older adults. It also represents a substantial global public health burden due to its high prevalence and impact on quality of life [1]. The burden of the disease is expected to increase further with population aging and AMD continues to be an important cause of visual disability and reduced quality of life in the elderly population. The disease primarily involves the macula and affects central vision, thereby limiting daily activities, including reading, driving, and recognizing faces. In both clinical practice and research, best-corrected visual acuity (BCVA) remains one of the most widely accepted indicators of functional status in AMD. It is simple, standardized, and clinically meaningful.

AMD is not only a degenerative disorder but also a multifactorial disease. Aging, oxidative stress, complement dysregulation, inflammation, and tissue remodeling are all involved in its pathogenesis, and these processes interact in a complex manner throughout disease development and progression [2]. Among these mechanisms, inflammation and blood-retinal barrier dysfunction have attracted increasing attention. The blood-retinal barrier is essential for maintaining retinal homeostasis, and its impairment may contribute to vascular leakage, inflammatory cell migration, and retinal tissue injury [3,4]. Claudin-5 is a major tight-junction protein of endothelial cells and plays an important role in barrier integrity and vascular permeability [5]. Experimental evidence indicates that claudin-5 dysregulation may compromise inner blood–retinal barrier integrity, promote vascular permeability, and contribute to retinal degenerative changes implicated in AMD pathogenesis [6]. Measurement of circulating tight-junction components has been proposed as a novel approach for evaluating blood–brain barrier dysfunction in several neurological and psychiatric disorders [7,8]. Given the structural and functional similarities between the blood–brain barrier and the inner blood–retinal barrier, comparable mechanisms may also be relevant in retinal diseases [9]. Claudin-5, a key endothelial tight-junction protein involved in barrier integrity and vascular permeability, was therefore evaluated as an exploratory peripheral biomarker in AMD [7,8]. In parallel, interest in systemic inflammatory biomarkers has also increased in retinal diseases. Easily obtainable hemogram-derived indices such as the neutrophil-to-lymphocyte ratio (NLR), platelet-to-lymphocyte ratio (PLR), monocyte-to-lymphocyte ratio (MLR), and pan-immune-inflammation value (PIV) are considered practical indicators of systemic inflammatory status. Several studies have examined especially NLR and PLR in AMD, and some reports have suggested that these markers may be elevated in neovascular disease [10,11,12]. However, the findings have not been fully consistent across studies. Because AMD is a localized retinal disorder, whether peripheral measurements of barrier-related proteins can meaningfully reflect disease phenotype remains uncertain. Nevertheless, investigating circulating claudin-5 together with systemic inflammatory indices may help determine whether a measurable peripheral signal accompanies AMD phenotypes. Therefore, the aim of this study was to evaluate serum claudin-5 levels and hemogram-derived inflammatory indices in patients with wet and dry AMD and to explore their associations with visual function.

## 2. Materials and Methods

This prospective case–control study was conducted in 2025 at the Ophthalmology Outpatient Clinic of Fethi Sekin City Hospital. The study population consisted of 60 patients diagnosed with age-related macular degeneration (AMD), including 30 patients with exudative (wet) AMD and 30 patients with non-exudative (dry) AMD. In addition, 30 healthy volunteers without AMD or any ocular pathology were included as the control group. The sample size was determined based on an a priori power analysis performed during the ethics application process using G*Power software, version 3.1.9.7 (Heinrich Heine University Düsseldorf, Düsseldorf, Germany).

Since no previously published human data were available regarding serum Claudin-5 levels, a formal power analysis based on effect size could not be performed. For this reason, the study was planned as an exploratory and hypothesis-generating investigation. The sample size was determined by taking into account the sample ranges of similar clinical studies evaluating serum biomarkers in AMD, together with the feasibility of the study. The study was conducted in line with the principles of the Declaration of Helsinki. Ethical approval was obtained from the Ethics Committee of Fethi Sekin City Hospital (Protocol No: 2025/11-40; Date: 12 June 2025). All participants provided written informed consent prior to enrollment.

All participants underwent a detailed ophthalmological examination according to a standardized protocol. Best-corrected visual acuity (BCVA) was measured using a Snellen chart. These values were converted to logMAR units for statistical analysis. Anterior segment findings were evaluated by slit-lamp biomicroscopy (Topcon Corporation, Tokyo, Japan). Optical coherence tomography (OCT-HS100 Canon Inc., Tokyo, Japan) was performed in all participants to assess posterior segment morphology and to differentiate AMD phenotypes. Fundus fluorescein angiography (FFA) was performed when choroidal neovascularization was suspected or when additional vascular assessment was required for diagnostic confirmation.

Patients aged 50 years or older who were diagnosed with wet or dry AMD based on clinical ophthalmic examination and multimodal imaging findings were eligible for inclusion in the study. The wet AMD group included patients with neovascular AMD diagnosed on clinical examination and multimodal imaging, whereas the dry AMD group included patients with non-exudative AMD. Patients who had received intravitreal anti-VEGF injection within the previous 6 months were excluded. The control group consisted of individuals without AMD or any other ocular pathology on detailed ophthalmic examination and OCT. In order to minimize the possible effects of confounding factors on systemic inflammatory markers and hematological parameters, individuals with inflammatory, autoimmune, hematological, or oncological diseases were excluded from the study. Patients with major organ dysfunction were also not included. In addition, ocular diseases other than AMD that might affect the retinal vascular microenvironment, a history of ocular surgery or intravitreal injection within the previous 6 months, and the use of systemic immunomodulatory therapy were accepted as exclusion criteria. To avoid inter-eye correlation, only one eye per participant was included in the analysis; when both eyes were eligible, the eye with the poorer BCVA was selected.

### 2.1. Biochemical and Hematological Analyses

All venous blood samples were collected between 08:00 and 10:00 AM. In this way, the possible effect of circadian variation on circulating biomarker levels was minimized. Serum samples were stored at −80 °C until analysis, in accordance with the manufacturer’s instructions. Hemolyzed samples were excluded from the analysis. Complete blood count parameters were analyzed using an automated hematology analyzer (Beckman Coulter UniCel DxH 800; Brea, CA, USA). Neutrophil, lymphocyte, monocyte, and platelet counts were recorded for all participants. Based on these values, NLR, PLR, MLR were calculated as the neutrophil-to-lymphocyte, platelet-to-lymphocyte, and monocyte-to-lymphocyte ratios, respectively. PIV was determined by multiplying the neutrophil, platelet, and monocyte counts and then dividing the product by the lymphocyte count.

Venous blood claudin-5 levels were measured by ELISA according to the manufacturer’s instructions using a commercially available kit (SunRed Bio, Shanghai, China; Lot No. 202505). The assay range of the kit was 0.15–30 ng/mL, and the analytical sensitivity was 0.116 ng/mL. According to the manufacturer, intra- and inter-assay CVs were <10% and <12%, respectively. Plate washing procedures were performed using a CombiWash automated microplate washer (Human Gesellschaft für Biochemica und Diagnostica mbH, Wiesbaden, Germany). Absorbance measurements were obtained using ChroMate and Microplate Reader P4300 devices (Awareness Technology Instruments, Palm City, FL, USA).

### 2.2. Statistical Analysis

Statistical evaluation was carried out using IBM SPSS Statistics version 22.0 (IBM Corp., Armonk, NY, USA). The normality of data distribution was examined with the Shapiro–Wilk test. Since the data were not normally distributed, continuous variables were presented as median (interquartile range, IQR), and categorical variables were expressed as number and percentage.

Comparisons among the three groups were performed using the Kruskal–Wallis test for continuous variables and the chi-square test for categorical variables. For parameters showing a significant overall difference, pairwise post hoc comparisons were performed using the Mann–Whitney U test with Bonferroni correction. For these post hoc comparisons, the corrected level of statistical significance was accepted as *p* < 0.0167. Correlations between serum claudin-5 levels and other clinical and laboratory parameters were evaluated using Spearman correlation analysis. In addition, linear regression analysis was performed to further evaluate the possible effect of age on serum claudin-5 levels. A two-tailed *p* value < 0.05 was considered statistically significant.

## 3. Results

### 3.1. Demographic and Clinical Characteristics

The demographic and clinical characteristics of the study groups are presented in Table 1. A total of 90 individuals were included in the study, comprising 30 healthy controls, 30 patients with wet age-related macular degeneration (AMD), and 30 patients with dry AMD. Age differed among the groups (*p* = 0.032), with the highest median age observed in the dry AMD group. Sex distribution was comparable across the groups (*p* = 0.551). BCVA (logMAR) also differed among the groups (*p* < 0.001), being highest in the wet AMD group, followed by the dry AMD group and controls. Post hoc analysis showed higher BCVA values in both AMD groups than in controls (both *p* < 0.001). The wet AMD group also had numerically higher BCVA values than the dry AMD group, but this difference was not significant after Bonferroni correction (*p* = 0.019; corrected threshold: *p* < 0.0167).

#### 3.1.1. Laboratory Findings and Inflammatory Indices

**Figure 1:**
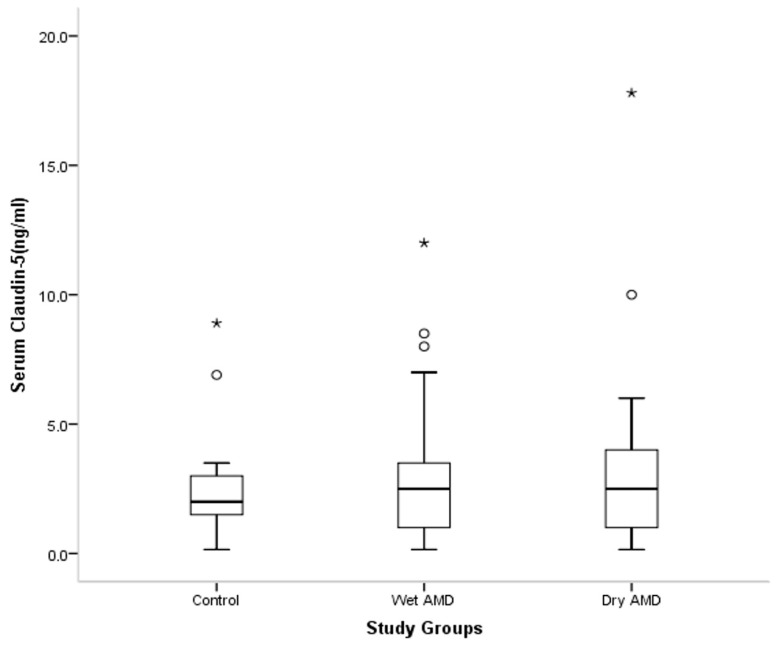
Distribution of serum Claudin-5 levels among the study groups. Data are presented as box-and-whisker plots showing median, interquartile range, whiskers, and outliers. Circles indicate outliers, and asterisks indicate extreme outliers. Kruskal–Wallis test: *p* = 0.280.

**Figure 2:**
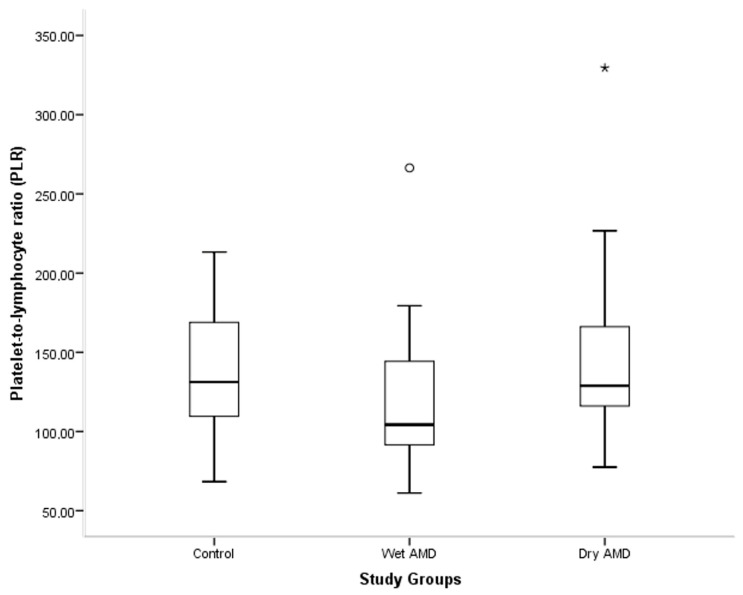
Distribution of PLR values among the study groups. Data are presented as box-and-whisker plots showing median, interquartile range, whiskers, and outliers. Circles indicate outliers, and asterisks indicate extreme outliers. Kruskal–Wallis test: *p* = 0.019.

Laboratory and inflammatory markers are summarized in Table 2. Median serum claudin-5 levels were 2.42 in controls, 3.28 in the wet AMD group, and 3.10 in the dry AMD group, with no difference among the groups (*p* = 0.280) (Figure 1). NLR values were likewise comparable among the groups (*p* = 0.310). By contrast, PLR differed among the groups (*p* = 0.019), with the lowest median value observed in the wet AMD group. Post hoc analysis showed that PLR was higher in the dry AMD group than in the wet AMD group (*p* = 0.013). To further assess the possible effect of age on PLR, linear regression analysis was performed. Age was not a significant predictor of PLR (*p* = 0.578). The difference between the dry and wet AMD groups was no longer statistically significant after adjustment for age (Bonferroni-adjusted *p* = 0.098). MLR and PIV did not differ among the groups (*p* = 0.410 and *p* = 0.752, respectively) (Figure 2).

#### 3.1.2. Correlation and Regression Analyses

Correlation analysis of Claudin-5 is presented in Table 3. No significant correlation was found between Claudin-5 and age (r = −0.070, *p* = 0.510), NLR (r = 0.089, *p* = 0.402), PLR (r = −0.002, *p* = 0.984), MLR (r = 0.174, *p* = 0.102), PIV (r = 0.121, *p* = 0.256), or BCVA (logMAR) (r = 0.047, *p* = 0.660). To further evaluate the possible effect of age, linear regression analysis was performed with serum Claudin-5 as the dependent variable and age and study group variables as independent variables. Neither age (*p* = 0.122) nor study group status (wet AMD vs. control, *p* = 0.214; dry AMD vs. control, *p* = 0.153) was significantly associated with circulating Claudin-5.

## 4. Discussion

In the present study, circulating claudin-5 levels did not differ significantly among the control, wet AMD, and dry AMD groups and were not associated with age, BCVA, or systemic inflammatory indices. Among the inflammatory markers, only PLR differed between AMD phenotypes, whereas NLR, MLR, and PIV were similar across the groups. Overall, these findings suggest that a single peripheral serum measurement of claudin-5 may have limited ability to reflect AMD phenotype, visual status, or the systemic inflammatory profile, and may not adequately capture local retinal barrier-related alterations. Rather than arguing against a role for claudin-5 in AMD biology, the lack of a significant between-group difference may indicate that local retinal barrier-related alterations are not easily captured in peripheral circulation. In addition, the BCVA differences observed between the AMD groups and controls, as well as between wet and dry AMD, support the expected clinical distinction between these phenotypes. Patients with dry AMD were older than those in the other groups. This may partly reflect the natural course of the non-neovascular phenotype, although the cross-sectional design precludes firm interpretation [13]. Moreover, age was not significantly associated with circulating claudin-5, suggesting that the absence of a between-group difference is unlikely to be explained solely by age.

These findings should be interpreted in the context of the existing literature on blood-retinal barrier dysfunction in AMD. Claudin-5 is one of the main tight-junction proteins of the inner blood-retinal barrier and plays an important role in endothelial integrity and vascular permeability [14,15]. Experimental studies suggest that claudin-5 dysregulation may compromise retinal barrier integrity and increase vascular permeability, thereby contributing to retinal dysfunction or injury [16]. Despite this biological background, we did not detect a significant difference in circulating claudin-5 among AMD phenotypes. This finding may suggest that claudin-5 dysregulation in AMD is mainly a local event at the retinal or choroidal level and may not be directly reflected in peripheral blood. Similar findings have been reported in blood–brain barrier-related disorders, where peripheral claudin-5 and occludin levels have shown heterogeneous results across studies [17]. This supports the view that local retinal barrier-related alterations in AMD may not be readily captured by single-point peripheral serum measurements and may remain largely confined to the retinal microenvironment [17,18]. Nevertheless, peripheral blood analysis of claudin-5 may still be of exploratory value as a minimally invasive biomarker approach. The absence of a significant serum difference in the present study may suggest that local retinal or choroidal barrier-related changes are not readily detectable by a single peripheral blood measurement. This should not be interpreted as evidence against the biological relevance of claudin-5 to blood-retinal barrier integrity in AMD.

Another important finding of the present study was the lack of a significant relationship between circulating claudin-5 and BCVA. Although BCVA is a major functional parameter in AMD, visual acuity may be influenced by several structural retinal changes associated with the disease, including drusen formation, retinal pigment epithelium alterations, and neovascular activity [19]. Therefore, a direct relationship between a peripheral barrier-related biomarker and visual function may not always be evident. Similarly, the absence of a correlation between claudin-5 and routine inflammatory indices suggests that barrier-related alterations and systemic inflammation may represent different aspects of AMD pathophysiology. AMD is increasingly accepted as a multifactorial disease involving local innate immune activation, complement dysregulation, oxidative stress, and tissue remodeling [20,21]. The positive correlations among inflammatory indices, despite their lack of association with claudin-5, suggest that these indices may reflect a common systemic inflammatory background rather than local barrier-related changes.

Regarding the systemic inflammatory indices, our findings are partly consistent with previous studies, but not completely. A systematic review and meta-analysis reported that NLR was generally higher in AMD than in controls, particularly in neovascular AMD [22]. Some previous studies also found increased NLR and PLR values in wet AMD and reported associations between these indices and visual function [12]. However, the literature is not fully consistent. Other reports have shown that routine hemogram-derived inflammatory indices do not always clearly distinguish AMD subtypes or separate wet AMD from controls [23]. Our findings appear to be closer to this more cautious interpretation. Differences among studies may be attributed to variations in sample size, patient selection, disease phenotype, treatment status, and the known susceptibility of hemogram-derived inflammatory indices to systemic and pre-analytical factors.

In the unadjusted analysis, PLR was the only inflammatory index that differed among the groups, with higher values in dry AMD than in wet AMD. However, this difference was attenuated and no longer statistically significant after adjustment for age. This suggests that the observed unadjusted PLR pattern may have been influenced, at least in part, by age distribution rather than reflecting an independent association with AMD phenotype. Similar inconsistency has also been reported in previous studies, in which PLR did not show a stable or independent association with AMD-related outcomes [23]. Therefore, the PLR finding in the present study should be interpreted with caution, and further studies are needed before drawing firm conclusions. Similarly, the MLR and PIV were not significantly associated with AMD phenotype in the present study. Nevertheless, previous reports have described increased MLR and PIV values in AMD, indicating that the relationship between these hemogram-derived inflammatory indices and AMD may not be consistent across cohorts [24,25,26]. Such discrepancies may reflect differences in study design, population characteristics, disease classification, and control of confounding variables.

Our findings may have two main implications. First, routine systemic inflammatory indices should be interpreted cautiously when used as phenotyping tools in AMD, especially if they are evaluated alone. Second, while claudin-5 remains a fundamental molecular player in barrier integrity, its circulating levels appear insufficient as a stand-alone peripheral surrogate for distinguishing AMD phenotypes or reflecting functional impairment. The novelty of the present study lies in the combined evaluation of circulating claudin-5 and hemogram-derived inflammatory indices across wet AMD, dry AMD, and control groups. Its significance is that it helps clarify both the potential and the limitations of peripheral biomarker analysis in a localized retinal disease, particularly with respect to blood-retinal barrier-related biology. From a translational perspective, these findings suggest that systemic measurements alone may not be sufficient. Integrative approaches combining systemic biomarkers with multimodal imaging and intraocular fluid biomarkers may provide a more comprehensive assessment of disease activity, particularly in capturing barrier dysfunction, inflammation, and angiogenic processes [27].

Several limitations of this study should be acknowledged. First, the sample size was relatively small, which may have reduced statistical power, particularly for subgroup and correlation analyses. Second, the cross-sectional design and single-time-point serum measurement do not allow causal or temporal interpretation. Third, circulating claudin-5 was measured in peripheral blood and may not fully reflect local retinal barrier alterations [28]. Intraocular fluid measurements were not available in the present study, and parallel assessment of aqueous or vitreous biomarkers together with serum levels might have provided a more direct evaluation of whether local barrier-related alterations are reflected in peripheral circulation. Fourth, the groups were not fully age-matched; possible heterogeneity within the wet and dry AMD groups, as well as prior treatment history, may have influenced the results. In addition, systemic comorbidities and other non-AMD-related confounders, such as smoking, alcohol consumption, and chronic medication use, may have affected the inflammatory indices. Fifth, the absence of concurrent imaging-based markers limits mechanistic interpretation. Finally, this was a single-center study, which may limit the generalizability of the findings.

## 5. Conclusions

In conclusion, circulating claudin-5 did not differ significantly across AMD phenotypes and was not associated with age, BCVA, or systemic inflammatory indices. Although PLR differed between AMD phenotypes, these findings suggest that single-point peripheral serum measurements of claudin-5 may have limited utility in reflecting local retinal barrier-related changes in AMD. Larger longitudinal studies, ideally incorporating intraocular and systemic biomarker assessments, are needed to clarify its potential biomarker value.

## Figures and Tables

**Table 1:** Demographic characteristics of the study groups.

Variable	Control (*n* = 30)	Wet AMD (*n* = 30)	Dry AMD (*n* = 30)	*p* Value
Age (years)	66.0 (59.3–74.0)	67.5 (63.0–76.3)	72.5 (68.8–76.0)	0.032
Male/Female, *n*	17/13	20/10	16/14	0.551
BCVA (logMAR)	0.00 (0.00–0.01)	1.00 (0.70–1.30)	0.61 (0.30–1.00)	<0.001

Values are presented as median (Q1–Q3) for age and number (percentage) for sex. Age differences between groups were analyzed using the Kruskal–Wallis test, and sex distribution was compared using the chi-square test. AMD: age-related macular degeneration; BCVA: best-corrected visual acuity.

**Table 2:** Laboratory and inflammatory markers of the study groups.

Parameter	Control (*n* = 30)	Wet AMD (*n* = 30)	Dry AMD (*n* = 30)	*p* Value
Claudin-5	2.42 (1.20–3.80)	3.28 (1.40–4.60)	3.10 (1.30–4.40)	0.28
NLR	2.15 (1.77–2.88)	1.87 (1.59–2.47)	2.47 (1.81–3.75)	0.31
PLR	131.22 (99.19–161.24)	104.32 (83.47–137.11)	128.85 (109.30–161.79)	0.019 *
MLR	0.31 (0.25–0.37)	0.30 (0.24–0.35)	0.33 (0.26–0.40)	0.41
PIV	332.92 (227.63–470.06)	262.18 (206.06–457.55)	336.64 (217.23–499.10)	0.752

Data are shown as median (interquartile range). Intergroup comparisons were performed with the Kruskal–Wallis test, followed by Mann–Whitney U tests with Bonferroni adjustment for post hoc analysis where appropriate. * PLR differed significantly between the dry and wet AMD groups (*p* = 0.013). AMD, age-related macular degeneration; NLR, neutrophil-to-lymphocyte ratio; PLR, platelet-to-lymphocyte ratio; MLR, monocyte-to-lymphocyte ratio; PIV, pan-immune-inflammation value.

**Table 3:** Correlation analysis of Claudin-5.

Variable	r	*p*
Age	−0.070	0.510
NLR	0.089	0.402
PLR	−0.002	0.984
MLR	0.174	0.102
PIV	0.121	0.256
BCVA (logMAR)	0.047	0.660

BCVA, best-corrected visual acuity; NLR, neutrophil-to-lymphocyte ratio; PLR, platelet-to-lymphocyte ratio; MLR, monocyte-to-lymphocyte ratio; PIV, pan-immune-inflammation value.

## Data Availability

All data generated or analyzed during this study are included in this article. Further details are available from the corresponding author upon reasonable request.
